# The relationship between microvascular complications and vitamin D deficiency in type 2 diabetes mellitus

**DOI:** 10.1186/s12902-015-0029-y

**Published:** 2015-06-25

**Authors:** Celil Alper Usluogullari, Fevzi Balkan, Sedat Caner, Rifki Ucler, Cafer Kaya, Reyhan Ersoy, Bekir Cakir

**Affiliations:** Clinic of Endocrinology and Metabolism, Atatürk Training and Research Hospital, Ankara, Turkey

**Keywords:** Type 2 diabetes mellitus, 25-hydroxyvitamin D, Microvascular complications

## Abstract

**Background:**

Vitamin D deficiency is reported as a possible risk factor for the development of diabetes in several epidemiologic studies. In this study, we investigated the frequency of 25-OH vitamin D deficiency in type 2 diabetes mellitus and the relationship between 25-OH vitamin D deficiency and the prevalence of microvascular complications.

**Methods:**

In this retrospective study, we evaluated the medical records of 557 patients with type 2 diabetes admitted to the Endocrinology Outpatient Clinic from January to March 2010 and 112 healthy controls randomly selected from individuals admitted to the hospital for a check-up and who had a laboratory result for serum 25-OH vitamin D concentrations at screening. The levels of 25-OH vitamin D in patients with type 2 diabetes and the relationship between 25-OH vitamin D deficiency and microvascular complications were investigated.

**Results:**

No significant difference in serum 25-OH vitamin D concentrations was observed between the diabetic and control groups. No correlation was observed between HbA_1C_ and serum 25-OH vitamin D levels. Serum 25-OH vitamin D levels were lower in diabetic patients with nephropathy, and patients not using any medication, i.e., those treated with dietary changes alone, had a higher prevalence of nephropathy.

**Conclusion:**

Vitamin D deficiency is more common in diabetic patients with nephropathy. When microvascular complications were evaluated, vitamin D levels were found to be lower in patients in whom these complications were more severe. Vitamin D deficiency is therefore associated with microvascular complications in diabetic patients.

## Background

Vitamin D deficiency is an important risk factor for glucose intolerance [1]. Studies have shown impaired insulin synthesis and secretion in animal models with vitamin D deficiency; diabetes onset can be delayed with 1–25-OH vitamin D intake, and some specific studies have reported that vitamin D deficiency contributes to the etiology and progression of type 2 diabetes [2, 3]. 25-OH vitamin D concentrations were found to be lower in patients with type 2 diabetes with impaired glucose tolerance than in controls [4]. Vitamin D is a known suppressor of renin biosynthesis, and vitamin D deficiency has been associated with progression of chronic kidney disease (CKD). Patients with type 2 diabetes and CKD have an exceptionally high rate of severe 25-OH vitamin D deficiency. This study investigates a possible relationship between vitamin D status and microvascular complications in patients with type 2 diabetes.

## Methods

Patients with type 2 diabetes admitted to the Endocrinology Outpatient Clinic from January to March 2010 owing to dietary or medication needs were included in the study group, and healthy nondiabetic individuals admitted to the hospital for a general check-up and whose serum 25-OH vitamin D concentrations had been requested formed the control group.

The vitamin D status of patients and controls was screened retrospectively. Due to seasonal changes, only patients whose vitamin D status was assessed during the January to March period were included in the study. The 25-0H vitamin D data were evaluated retrospectively, so measurements for the other months could not be retrieved. Data were obtained from their medical records and included detailed histories, physical examination records, and laboratory findings (gender, age, height, weight, body mass index (BMI), waist circumference, fasting blood glucose, creatinine, HbA_1c_, low-density lipoprotein (LDL) cholesterol, high-density lipoprotein (HDL) cholesterol, triglycerides, and microalbumin levels in 24-h urine). Glomerular filtration rate (GFR) is the best overall index of kidney function. Normal GFR varies according to age, sex, and body size, and declines with age. The National Kidney Foundation recommends using the CKD Epidemiology Collaboration (CKD-EPI) Creatinine Equation (2009) to estimate GFR.

The medications received were stratified as diet alone, oral hypoglycemic agents, or insulin. The relationships between 25-OH vitamin D, HbA_1c_, microvascular complications of diabetes (nephropathy, retinopathy, and neuropathy), and type of treatment were investigated. Patients with CKD, active infection, liver disease, primary hyperparathyroidism, macroalbuminuria, osteoporosis, serum creatinine level > 2 mg/dL, or those receiving vitamin D or calcium supplements were excluded from the study. For a diagnosis of nephropathy, patients were defined as having microalbuminuria with 24-h urine albumin content of 30–300 mg/day. For diagnosing diabetic retinopathy (DR), all participants underwent an ophthalmic examination by an experienced ophthalmologist. This examination consisted of funduscopy by slit-lamp with 90 D lenses and indirect ophthalmoscopy. DR was defined as the presence of one or more of the following lesions: microaneurysms, blot or flame-shaped hemorrhages, hard exudates, cotton-wool spots, or evidence of laser treatment for DR at baseline. DR was categorized as nonproliferative (NPDR) or proliferative (PDR), determined by the presence of retinal neovascularization.

Peripheral neuropathy was defined by abnormalities in monofilament testing, performed with a Semmes-Weinstein monofilament of 5.07/10 g, three times at each site (dorsal between the base of digits 1–2; ventral digits 1, 3, 5; metatarsal heads 1, 3, 5; medial and lateral midfoot; and heel). If the patient missed perceiving the filament more than once at one site, the test was considered abnormal at that site. If a subject did not perceive the filament at two or more of the 10 sites, the test was reported as abnormal. Vibration sensation was examined using a diapason on the dorsum of the big toe. A 128-Hz tuning fork was used to examine vibration perception at the dorsum of the interphalangeal joint of the right hallux. The vibrating tuning fork was placed on the interphalangeal joint and if nothing was felt, the score was two points. When some vibration was sensed, the still-vibrating tuning fork was immediately placed at the dorsal wrist. When the vibration felt the same as that at the hallux, the score was zero points, and when it felt stronger, the score was one point.

The study group consisted of 557 patients who were receiving dietary or medical treatment for confirmed type 2 diabetes. The control group consisted of 112 healthy subjects who were admitted for check-up. The study was conducted in accordance with International Conference on Harmonization Good Clinical Practice (ICH-GCP) guidelines, and the Declaration of Helsinki, and approval for the study was granted by the Ethical Committee of Ataturk Training and Research Hospital.

### Laboratory analysis

Owing to hospital regulations, antecubital vein blood sampling was performed between 8:00 and 10:00 a.m. on all subjects, healthy or with diabetes, after a 12-h fast. Fasting blood glucose was measured using a glucose oxidase method (Roche Diagnostic GmbH). Total cholesterol, HDL cholesterol, and triglycerides were measured by enzymatic methods (Boehringer, Mannheim, Germany). LDL concentrations were calculated via the Friedewald formula. 25-OH vitamin D concentration and HbA_1c_ were measured by a high-performance liquid chromatography (HPLC) method and by HPLC-ultraviolet (UV) detection, respectively.

### Statistical analysis

Statistical Package for Social Sciences (SPSS) 17.0 for Windows software (SPSS Inc, Chicago, IL, USA) was used for the data analysis. Continuous variables with a normal distribution are presented as mean ± standard deviation; non-normally distributed continuous variables are presented as median (minimum to maximum); ordinal variables are presented as median and mode; and nominal variables are presented as number and percentage. The normality of the distribution of the continuous variables was evaluated using histograms and the one-sample Kolmogorov-Smirnov test; distributions with p > 0.05 were accepted as normally distributed. The differences between normally distributed continuous independent variables were evaluated with the independent samples *t* test, and those without a normal distribution were evaluated with the Mann–Whitney *U* test. All hypotheses were examined using two-tailed tests and p < 0.05 was used as the significance level. The relationships between nominal variables were evaluated using the Pearson chi-squared test and the Fisher exact test; p < 0.05 was used as the significance level. Statistical significance of risk factors for complications in diabetic patients, the results obtained via univariate analysis were subjected to multivariate logistic regression.

## Results

The demographic characteristics of the subjects in the study are shown in Table 1. There were 296 men in the diabetic group and 45 in the control group. There were 261 women in the diabetic group and 67 in the control group. Mean (±SD) age in the diabetic and control groups was 55.2 ± 10.9 and 53.1 ± 12.5 years, respectively; a statistically significant difference between the groups was not observed (p = 0.07). Mean BMI in the diabetic and control groups was 30.3 ± 5.5 and 29.8 ± 5.7 kg/m^2^, respectively. The average duration of diabetes in the diabetic group was 5 years (0–20 years). The two groups were similar in terms of age, height, weight, and BMI (Table 1). HbA_1c_ levels were higher in the diabetic group than in controls, as expected (p < 0.001), and 25-OH vitamin D concentrations did not differ between the two groups (p = 0.302). Mean serum 25-OH vitamin D concentrations were higher in men than in women (21.89 ± 8.74 vs 20.24 ± 8.53 ng/mL, respectively; p = 0.025).

**Table 1:** Patient characteristics

	Diabetes group	Control group	*p* value
	(n = 557)	(n = 112)	
Gender (female/male), n	261/296	67/45	0.008
Age (years), mean ± SD	55.2 ± 10.9	53.09 ± 12.5	0.07
Height (m), mean ± SD	163.5 ± 9.7	163.1 ± 9.7	0.712
Weight (kg), mean ± SD	81.1 ± 15.1	78.8 ± 14.2	0.209
BMI (kg/m^2^), mean ± SD	30.3 ± 5.5	29.8 ± 5.7	0.381
HbA_1c_ (%), mean ± SD	7.7 ± 1.7	5.6 ± 0.3	<0.001
25-OH vitamin D (ng/mL), mean ± SD	21.1 ± 8.6	21.4 ± 11.8	0.302
GFR (mL/min per 1.73 m^2^), mean ± SD	78.25 ± 21.5	80.1 ± 21.3	0.416

*BMI* body mass index, *HbA*_*1c*_ glycated hemoglobin, *GFR* glomerular filtration rate

Table 2 summarizes the prevalence of various microvascular complications observed in both groups. Microvascular complications were present in 172 of 555 patients (30.9 %). The mean 25-OH vitamin D concentrations in the groups with or without microvascular complications were estimated to be 19.1 ± 8.1 and 22.0 ± 8.7 ng/mL, respectively; this difference was statistically significant (p < 0.001). In the diabetic group, nephropathy was observed in 91 patients (16.3 %). The mean 25-OH vitamin D concentrations in the groups with or without nephropathy were estimated to be 18.52 ± 8.2 and 21.6 ± 8.6 ng/mL, respectively. Patients with type 2 diabetes had lower total 25-OH vitamin D concentrations than patients with any of the microvascular complications (p < 0.001) or patients with nephropathy (p = 0.002). In the diabetic group, retinopathy was observed in 73 of 311 patients (23.4 %). Similar 25-OH vitamin D levels were observed in both groups, with or without retinopathy (20.0 ± 7.7 vs 19.7 ± 8.4 ng/mL; p = 0.653). In the diabetic group, neuropathy was observed in 89 of 311 patients (28.6 %). The mean 25-OH vitamin D concentrations in the groups with or without neuropathy were estimated to be 19.4 ± 7.9 and 20.0 ± 8.4 ng/mL, respectively; this difference was not statistically significant (p = 0.617).

**Table 2:** Mean 25-OH vitamin D levels in patients with type 2 diabetes and microvascular complications

		Number of patients	25-OH vitamin D (ng/mL)	*p* value
			Mean ± SD	
**Microvascular complications**	**Present**	172	19.1 ± 8.1	**<0.001**
	**Absent**	383	22.0 ± 8.7	
Nephropathy	Present	84	18.52 ± 8.2	**0.002**
	Absent	466	21.6 ± 8.6	
Retinopathy	Present	73	20.0 ± 7.7	0.653
	Absent	238	19.7 ± 8.4	
Neuropathy	Present	89	19.4 ± 7.9	0.617
	Absent	222	20.0 ± 8.4	

Table 3 presents a comparison of the patient and control groups where 25-OH vitamin D concentrations were either above or below the threshold value of 20 ng/mL. 25-OH vitamin D levels <20 ng/mL and ≥20 ng/mL were observed in 101 and 71 patients with microvascular complications, respectively; the difference between the groups was statistically significant (p < 0.001, *χ*^2^ = 17.3). 25-OH vitamin D levels of <20 ng/mL were observed in 53 of 84 patients with nephropathy; a statistically significant difference was observed between the groups (p < 0.001, *χ*^2^ = 12.7). 25-OH vitamin D levels of <20 ng/mL were observed in 40 of 73 patients with retinopathy; the difference between the groups was not statistically significant (p = 0.929, *χ*^2^ = 0.1). 25-OH vitamin D levels of <20 ng/mL were observed in 49 of 89 patients with neuropathy; the difference between the groups was not statistically significant (p = 0.873, *χ*^2^ = 0.1). The incidence of microvascular complications and nephropathy was higher in the patients with type 2 diabetes whose 25-OH vitamin D concentrations were <20 ng/mL compared with patients whose 25-OH vitamin D concentrations were ≥20 ng/mL.

**Table 3:** Comparison of patients with type 2 diabetes with 25-OH vitamin D levels below or above the 20 ng/mL threshold

		25-OH vitamin D		
		<20 ng/mL	≥20 ng/mL	*p* value
**Microvascular complications**	**Present**	101	71	**<0.001**
	**Absent**	152	231	***χ*** ^**2**^ **= 17.3**
Nephropathy	Present	53	31	**<0.001**
	Absent	196	270	***χ*** ^**2**^ **= 12.7**
Retinopathy	Present	40	33	0.929
	Absent	129	109	*χ* ^2^ = 0.1
Neuropathy	Present	49	40	0.873
	Absent	120	102	*χ* ^2^ = 0.1

There was no correlation between HbA_1c_ and serum 25-OH vitamin D levels (p = 0.095; r = −0.65), and HbA_1c_ levels were similar across groups where 25-OH vitamin D concentrations were less than, equal to, or more than 20 ng/mL (p = 0.167, Z = 1.38).

Table 4 shows the 25-OH vitamin D levels and complication rates in groups stratified by antidiabetic treatment received. In this study of 557 patients with type 2 diabetes, microvascular complications were observed in 6 of 18 (33.3 %) patients on diet only, 102 of 341 (29.9 %) patients on oral antidiabetic drugs, and 138 of 198 (69.6 %) patients on insulin treatment. However, nephropathy was significantly more prevalent in the group treated by diet alone than in those receiving any type of hypoglycemic medication (p < 0.001; *χ*^2^ = 51.4), and neuropathy was more prevalent in patients receiving insulin or oral hypoglycemic treatments (p = 0.008; *χ*^2^ = 9.6).

**Table 4:** 25-OH vitamin D levels and rates of diabetes-related complications by treatment received

	Diet only	Oral antidiabetic treatment	Insulin treatment
	(n = 18)	(n = 341)	(n = 198)
25-OH vitamin D (ng/dL), (mean ± SD)	20.63 ± 10.2	21.4 ± 8.2	20.9 ± 8.5
Complications n (%)	6 (33.3)	102 (29.9)	138 (69.6)
Nephropathy	5 (27.8)	34 (9.9)	45 (22.7)
Neuropathy	0 (0)	42 (12.3)	47 (23.7)
Retinopathy	1 (9.1)	26 (7.6)	46 (23.2)

Table 5 shows the 25-OH vitamin D levels and complication rates in patients with type 2 diabetes stratified by gender.

**Table 5:** 25-OH vitamin D levels and rates of diabetes-related complications by gender

	Male	Female	*p* value
	(n = 294)	(n = 261)	
25-OH vitamin D (ng/dL), (mean ± SD)	21.89 ± 8.7	20.24 ± 8.5	0.025
Complications, n (%)	103 (35)	69 (26.4)	0.034
Nephropathy	50 (17.2)	34 (13.1)	0.193
Neuropathy	50 (29.2)	39 (27.9)	0.802
Retinopathy	42 (24.6)	31 (22.1)	0.687

The risk factors for complications in patients with type 2 diabetes, based on both univariate and multivariate analyses, are shown in Table 6. Multivariate analyses showed that HBA_1C_ and 25-OH vitamin D were independent predictors of complications in patients with type 2 diabetes.

**Table 6:** Risk factors for complications in patients with type 2 diabetes, based on univariate and multivariate analysis

Risk factors	p value	Odds ratio	95 % CI
**Univariate analysis**			
Fasting blood glucose	<0.001		0.595**–**0.703
HbA_1c_	<0.001		0.637**–**0.743
LDL-cholesterol	0.126		0.396**–**0.514
Triglycerides	0.065		0.496**–**0.613
25-OH vitamin D	0.07		0.363**–**0.476
**Multivariate analysis**			
Fasting blood glucose	0.307	0.998	0.993**–**1.002
**HbA**_**1c**_	**0.006**	**1.275**	**1.073–1.516**
LDL-cholesterol	0.661	0.999	0.994**–**1.004
Triglycerides	0.063	1.002	1.000**–**1.003
**25-OH vitamin D**	**0.018**	**0.970**	**0.945–0.995**
**Age**	**0.400**	**1.009**	**0.993–1.025**
**BMI**	**0.639**	**0.991**	**0.955–1.029**
**Gender**	**0.081**	**1.447**	**0.955–2.191**

*BMI* body mass index, *HbA*_*1c*_ glycated hemoglobin, *LDL* low-density lipoprotein

## Discussion

The risk for diabetes and associated metabolic abnormalities increases with vitamin D deficiency [5–7]. Vitamin D status may influence the risk of developing metabolic diseases such as type 2 diabetes, metabolic syndrome, and insulin resistance [8].

Evidence from observational studies indicates inverse association of circulating 25-OH vitamin D with risk of death due to cardiovascular disease, cancer, or other causes [9]. Vitamin D deficiency rates are also reported to be higher among people with type 2 diabetes [10]. In their study of 825 patients undergoing dialysis, Wolf et al. [11] observed a vitamin D deficiency rate of 78 %, and this deficiency was associated with an increased early mortality risk; when 25-OH vitamin D was <10 ng/mL, the mortality rate increased 1.9-fold, and with values between 10 and 30 ng/mL, the mortality rate increased 1.4-fold. A meta-analysis by Pittas et al. [5] reported that vitamin D deficiency was common in diabetic patients, and diabetic complications could be delayed or prevented by vitamin D supplementation. In contrast to most reports, serum 25-OH vitamin D concentrations did not differ between subjects with or without diabetes in our study.

Selective vitamin D receptor activator paricalcitol effectively reduce proteinuria in patients with type 2 diabetes through inhibition of the renin-angiotensin-aldosterone system (RAAS) [12]. Preclinical studies have shown that vitamin D is renoprotective [13]. Paricalcitol therapy did not affect plasma N-terminal probrain natriuretic peptide concentration in patients with type 1 diabetes and diabetic nephropathy; however, the urinary albumin excretion rate was significantly lowered [14]. In a study conducted in China, vitamin D treatment reduced microalbuminuria in patients with type 2 diabetes and nephropathy. RAAS is known to play a major role in diabetic nephropathy, and vitamin D suppresses renin release. Vitamin D deficiency increases albuminuria in patients. Vitamin D replacement also has beneficial effects on other diabetic nephropathy risk factors, such as hypertension and hyperlipidemia [15, 16]. Vitamin D deficiency is highly prevalent in patients with advanced CKD, but diabetes, the most common cause of CKD, has also been linked to low levels of serum 25-OH vitamin D. The prevalence of 25-OH vitamin D deficiency (<20 ng/mL) was 70.8 % in patients with diabetes-related CKD, 38.8 % in patients with non-diabetic CKD, and 41 % in patients with diabetes without advanced CKD. Low vitamin D status is characteristically associated with advanced diabetic nephropathy [17]. The potential mechanisms of renal protection by vitamin D3 include inhibition of RAAS and protection of the kidney from inflammation, fibrosis, and structural changes. In addition, vitamin D3 treatment is not associated with significant adverse effects, including gastrointestinal adverse effects, and fluctuation of blood pressure. Vitamin D3 can ameliorate proteinuria and protect the kidney from injury in patients with diabetic nephropathy. This renoprotective effect is independent of blood pressure and glucose reduction and does not increase adverse effects over controls, even in combination therapy with angiotensin-converting enzyme inhibitors or angiotensin receptor blockers [18].

In the NHANES study [19], insulin resistance, kidney function, and vitamin D status of 14,679 patients were assessed, and vitamin D deficiency was reported to be associated with increased risks of microvascular and macrovascular complications in patients with type 1 as well as type 2 diabetes. In that study, 25-OH vitamin D concentrations were lower in patients with diabetes mellitus and nephropathy compared to patients without nephropathy. Decreased serum 25-OH vitamin D levels in people with severely decreased kidney function is independent of age, gender, ethnicity, and lifestyle variables known to affect vitamin D status, such as BMI, physical activity, and intake of dairy foods and vitamin D supplements. The relationship between serum 25-OH vitamin D concentration at baseline and the incidence of macrovascular (including myocardial infarction and stroke) and microvascular (retinopathy, nephropathy, neuropathy, and amputation) disease was analyzed. A 50 nmol/L difference in blood 25-OH vitamin D concentration was associated with a 23 % (p = 0.007) change in risk of macrovascular complications during the study; low serum 25-OH vitamin D concentrations were associated with an increased risk of macrovascular and microvascular disease events in type 2 diabetes [20].

Shebab et al. [21] showed that the onset of neuropathy can be delayed by vitamin D treatment. Vitamin D deficiency is more common in patients with distal symmetric polyneuropathy, and neuropathic pain has been reported to decline when the vitamin D deficiency is corrected [22, 23]. Several studies showed a possible association between vitamin D deficiency and diabetic peripheral neuropathy (DPN) in patients with type 2 diabetes. A meta-analysis showed that vitamin D deficiency was significantly associated with increased risk of DPN in patients with type 2 diabetes (odds ratio [OR] 2.88; 95 % CI, 1.84–4.50; p < 0.00001). A meta-analysis of three studies with adjusted estimates showed that vitamin D deficiency was independently associated with increased risk of DPN in patients with type 2 diabetes (OR 2.68; 95 % CI, 1.67–4.30; p < 0.0001) [24].

Vitamin D insufficiency is prevalent in patients with type 2 diabetes mellitus and is associated with peripheral neuropathy. However, there are few data regarding vitamin D status in patients with cardiovascular autonomic neuropathy. The mean circular resultant (MCR) (39.5 ± 26.3 vs 27.6 ± 17.2; p < 0.01) and the expiration/inspiration (E/I) ratio (1.21 ± 0.17 vs 1.15 ± 0.09; p < 0.01) were lower in patients with 25-OH vitamin D insufficiency after controlling for age. This suggests that 25-OH vitamin D insufficiency is associated with reduced parasympathetic function, with a stronger association in younger patients with type 2 diabetes [25]. Studies of patients with type 2 diabetes confirmed the relationship between vitamin D deficiency and neuropathy incidence as well as the severity of the symptoms caused by neuropathy. Recent studies also suggest a relationship between the incidence of plantar ulcers and vitamin D deficiency [26]. However, no associations between neuropathy and 25-OH vitamin D were observed in our study.

Several human studies have shown an inverse relationship between vitamin D levels and several chronic conditions associated with inflammation [27]. In the eye, vitamin D receptors are expressed extensively in the retina [28]. Therefore, vitamin D might prevent the development and progression of DR as a result of its anti-inflammatory and anti-angiogenic properties. There is emerging evidence that DR is initiated and propagated by inflammation and angiogenesis [29]. However, epidemiologic studies evaluating the relationship between vitamin D and DR are limited, although some clinic-based case–control studies have proposed a possible association between the two variables [30]. Vitamin D deficiency has been reported to be associated with increased prevalence of DR among young patients with type 1 diabetes; retinal vascular diseases were found to be associated with the inflammatory and angiogenic effects of vitamin D deficiency [31]. An inverse relationship was observed between the severity of retinopathy and the circulating 1,25-(OH) 2 D3 concentration among patients with type 2 diabetes, and a direct relationship was observed between lower 25-OH vitamin D concentration and the severity of the proliferative retinopathy [32]. A total of 204 patients with type 2 diabetes were subdivided into groups without diabetic retinopathy (NDR, n = 110) and those with DR (n = 94). Logistic analysis confirmed that the duration of diabetes (OR 1.108; p < 0.01), systolic blood pressure (OR 1.022; p < 0.05), and HbA_1c_ (OR 1.267; p < 0.05) were independently associated with the risk of DR [33]. The study found a sex-related difference in the association between serum 25-OH vitamin D levels and DR. Although men with high serum 25-OH vitamin D levels had 63 and 85 % lower risk of any DR and proliferative DR, respectively, relative to those with the lowest levels after adjusting for potential confounders, similar associations were not observed in women. Moreover, a sex-related effect modification was observed. The directions of the OR for any DR and proliferative DR in women (OR 1.58 and 1.99, respectively) were opposite to those in men (OR 0.37 and 0.15, respectively), which would explain the lack of an overall association. These findings suggest that vitamin D deficiency might be a significant risk factor for any DR and proliferative DR in men but not in women [34]. In one study, 1520 patients with type 2 diabetes were recruited and divided into three groups according to their fundus oculi results: no DR (n = 625, 41.12 %), non-sight-threatening DR (n = 562, 36.97 %), and sight-threatening DR (n = 333, 21.91 %). Vitamin D deficiency was defined as serum circulating 25-OH vitamin D level < 20 ng/mL. Vitamin D deficiency is an independent risk factor for DR and sight-threatening DR; the prevalence of sight-threatening DR doubles when the serum 25-OH vitamin D level is <15.57 ng/mL [35]. However, we found no significant associations between the vitamin D status and retinopathy in our present study.

## Conclusions

Previous reports have shown that a low concentration of serum 25-OH vitamin D increases the risk of developing diabetes later in life and is also associated with an increased risk of diabetic microvascular complications, though our study only showed such associations for nephropathy. The vitamin D status of patients with diabetes should be considered during their regular follow-up, and supplementation should be provided to those at risk of deficiency.

### Limitations of the study

Because this was as a retrospective study, all patient data could not be fully retrieved. There is a need for prospective or controlled studies with larger sample sizes where vitamin D replacement and complication regressions are followed and where the impact of the vitamin D levels on blood glucose regulation is observed.
